# Atypical Posterior Nutcracker Syndrome in a 17-Year-Old Male Without Hematuria

**DOI:** 10.7759/cureus.17221

**Published:** 2021-08-16

**Authors:** Mohamed Almuqamam, Mohamed Ebrahim, George Nassar, Matthew Kaplan

**Affiliations:** 1 Pediatrics, The Brooklyn Hospital Center, New York, USA; 2 Pediatric Nephrology, The Brooklyn Hospital Center, New York, USA

**Keywords:** renal nutcracker syndrome, retroaortic left renal vein, hematuria, renal colic, acute abdominal pain

## Abstract

Retro-aortic left renal vein (RLRV) is an anatomical variation, where the left renal vein (LRV) courses posterior to the aorta and anterior to the vertebrae before it eventually drains into the inferior vena cava (IVC). RLRV is a rare finding, with a prevalence of around 1%-2%, and only a small minority of RLRVs cause symptoms. RLRV symptoms occur secondary to compression of the LRV between the abdominal aorta and vertebrae, otherwise known as posterior nutcracker syndrome (PNCS). The most common symptoms of PNCS are hematuria and flank pain. We present a 17-year-old male, who came in with recurring left flank pain without hematuria, initially thought to be renal colic secondary to nephrolithiasis. On further investigations, an aberrant posterior renal vein was seen on CT suggestive of PNCS. The patient was treated successfully with RLRV vascular stent placement by interventional radiology. This case report adds to the limited number of PNCS cases observed in children and to the even rarer cases of PNCS without hematuria. This case also acts as a reminder for pediatricians to keep a wide scope of differentials in patients presenting with flank pain and provides an outline of both diagnostic and treatment modalities available for these patients.

## Introduction

Nutcracker syndrome (NCS) is an uncommon cause of hematuria that can be either microscopic or macroscopic and accompanied by flank pain. There are two different forms of NCS, anterior and posterior, which both produce similar symptomology, secondary to impaired outflow of the left renal vein (LRV) into the inferior vena cava (IVC).

Anterior NCS, the most common anatomical variant, occurs when the LRV courses anterior to the abdominal aorta but posterior to the superior mesenteric artery (SMA) resulting in compression of the LRV [1]. Posterior nutcracker syndrome (PNCS) is when the LRV follows a retro-aortic course and is compressed between the aorta and vertebral bodies. Compression of the LRV in either situation leads to renal venous hypertension. Over time, the high pressures can cause damage to the renal vasculature leading to hematuria [2]. In males, where the left testicular vein drains into the LRV, they can experience left-sided varicocele. In females, NCS can present as pelvic congestion syndrome (dysmenorrhea, dyspareunia, lower abdominal pain, and pelvic varices).

Intermittent flank pain that radiates to the posterior thigh and buttocks is another possible symptom [3]. NCS, however, is most commonly asymptomatic. Common differentials for hematuria and flank pain in the pediatric population include nephrolithiasis, pyelonephritis, acute interstitial nephritis, glomerular diseases, urinary tract infection, medications, trauma, and tumors [4].

## Case presentation

A 17-year-old male presented to the pediatrician with complaints of intermittent flank pain that began eight months prior. One month prior to attending our clinical practice for medical evaluation, the patient went to see his primary care physician who had prescribed painkillers, 10 days of antibiotics (Ciprofloxacin) and suggested increased fluid intake due to concerns for nephrolithiasis despite normal renal and bladder ultrasounds. The patient at that point was complaining of left flank pain, left anterior abdominal pain, and burning on urination. During the evaluation of the patient's medical history, the only significant finding was an older sister with kidney stones history, treated with lithotripsy.

Physical examination was significant of left costovertebral tenderness. Complete blood count and complete metabolic panel at initial presentation were normal with a creatinine of 0.8 mg/dL (0.6-1.2 mg/dL), urea 10 mg/dL (7-20 mg/dL), complete normal urinalysis, and negative urine culture as seen in Tables 1-3. 

**Table 1:** Results of the complete blood count.

Complete blood count	Result	Reference range
White blood count	5.5	4.5-13 Thous/mcL
Red blood count	5.3	4.10-5.70 Mill/mcL
Hemoglobin	16.2	12-16.9 g/dL
Platelets	188	140-400 Thous/mcL
Mean corpuscular volume	89.6	78-98 fL
Neutrophils %	52	40%-75%
Lymphocyte %	35.4	15%-50%

**Table 2:** Results of the complete metabolic panel.

Complete metabolic panel	Result	Reference range
Sodium	141	135-146 mmol/L
Potassium	4.5	3.8-5.1 mmol/L
Chloride	104	98-110 mmol/L
HCO3	28	20-31 mmol/L
Urea nitrogen	10	7-20 mg/dL
Creatinine	0.8	0.6-1.2 mg/dL
Calcium	10.1	8.9-10.4 mg/dL
Glucose (random)	88	65-139 mg/dl
Total protein	7	6.3-8.2 g/dL
Albumin	5	3.6-5.1 g/dL
Alkaline phosphatase	86	48-230 U/L
Aspartate amino transferase	16	12-32 U/L
Alanine amino transferase	15	8-46 U/L
Total bilirubin	0.8	0.2-1.1 mg/dL

**Table 3:** Results of urinalysis.

Urinalysis	Result	Reference range
Color	Yellow	Yellow
Appearance	Clear	Clear
Glucose	Negative	Negative
Bilirubin	Negative	Negative
Ketones	Negative	Negative
Specific gravity	1.023	1.001-1.035
Blood	Negative	Negative
Potential hydrogen	6.5	5-8
Protein	Negative	Negative
Nitrate	Negative	Negative
Leukocyte esterase	Negative	Negative
Squamous epithelial cells	None seen	< 5 cells/hpf
White blood count	None seen	< 5 cells/hpf
Red blood count	None seen	< 5 cells/hpf
Bacteria	None seen	None seen
Hyaline cast	None seen	None seen
Urine culture	Negative	Negative

**Figure 1:**
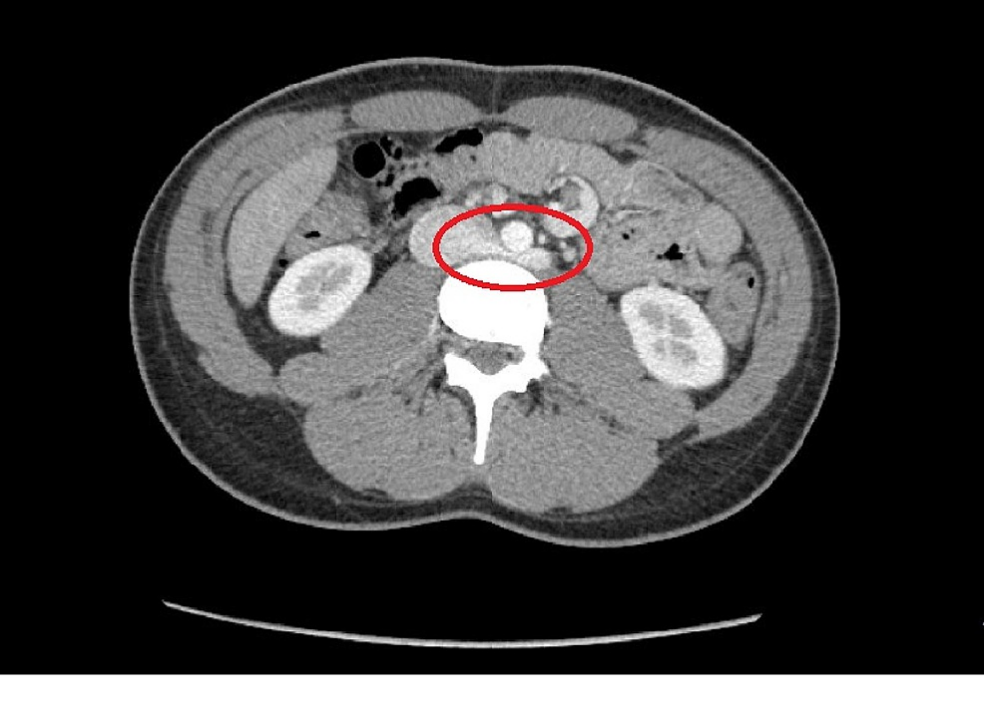
Axial contrast-enhancement CT of the abdomen at the level of the LRV outflow into the inferior vena cava. The red circle shows the abnormal retroarotic course of LRV, causing an anatomic compression between the aorta and the underlying vertebral body suggestive of posterior nutcracker syndrome LRV, left renal vein; IVC, inferior vena cava

Hence, an abdominal CT without contrast was done to look for renal stones, however, it did not reveal any stones but showed an aberrant RLRV suggestive of PNCS as seen in Figure 1.

**Figure 2:**
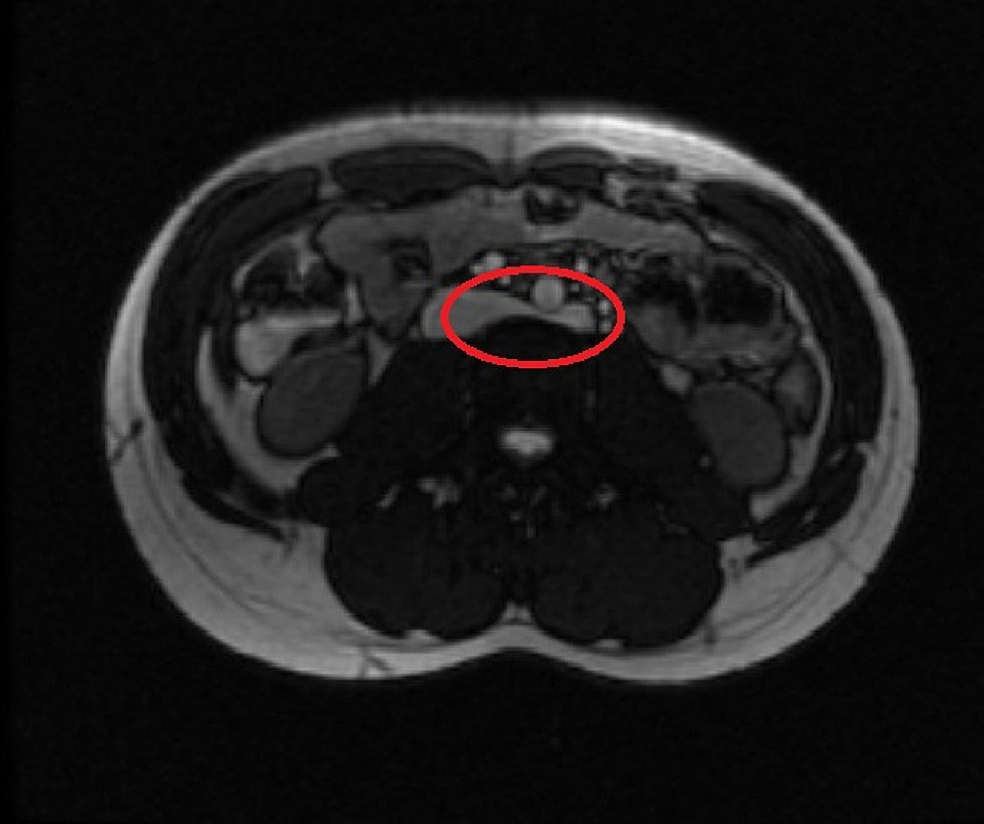
MRA of the abdomen on axial acquisition. The red circle reveals the retro-aortic left renal vein being compressed between the fifth lumbar vertebrae and the abdominal aorta MRA, magnetic resonance angiogram

Therefore, it was recommended that the patient followed up with a magnetic resonance angiogram (MRA) of the abdomen with IV pressure gradient measurements. Abdominal MRA confirmed a LRV that was compressed between the fifth lumbar vertebrae and the abdominal aorta. There was no evidence of hydronephrosis and no evidence of any renal mass. Figure 2 below shows the abdominal MRA findings.

The patient continued to have severe intermittent pain, so the decision was made to refer him for stent placement via interventional radiology. The procedure was a success and the patient became completely asymptomatic afterward.

## Discussion

The diagnosis of RLRV is often difficult, especially in pediatric patients, where it is often asymptomatic and easily overlooked on sonography. RLRVs are seen as vascular structures that communicate with the IVC, but course posteriorly to the aorta. When the space between the aorta and vertebra narrows, this can lead to PNCS. RLRV recognition is important in order to avoid complications in retroperitoneal surgeries as well as complications of PNCS, which include but are not limited to hematuria, varicocele, congested kidney, renal infarcts, renal vein thrombosis, ureteropelvic junction obstruction, bacterial localization, and abscess formation [2, 5].

The incidence of RLRV has been reported to be around 0.5%-2.3% [2-3] accounting for multiple variations of RLRV [1, 6-7]. RLRV occurs as an isolated finding in approximately 2% of the population [8]. It is important to consider RLRV compression in patients presenting with microscopic hematuria or left flank/abdominal pain. Younger infants with RLRV may present with a renal lump along with hematuria [4].

One of the major points of interest in our patient is the lack of classical hematuria both macroscopically and microscopically. Both anatomical variations of NCS have been classically described with hematuria, with the hematuria likely secondary to rupture of the small veins separating the septum of the urinary collecting system [9-10]. Case reports describing lack of blood in the urine, however, are scarce making this a rare presentation of an already rare syndrome [9]. NCS can be divided into two clinical variants based on the presence of hematuria. The first is the typical presentation (renal), these patients have hematuria, proteinuria, and flank pain and the second is the atypical presentation (urological) with no hematuria, flank pain, but the presence of abdominal pain, varicocele, dyspareunia, dysmenorrhea, and fatigue [10]. Our patient combined components of both clinical variants.

Due to the non-specific findings in PNCS, diagnostic workup may initially be based on the mode of patient presentation. Because microscopic hematuria in the pediatric population is generally benign, flank pain and hematuria usually are investigated initially with labs (urea, creatinine, complete blood count, and electrolytes), urinalysis and urine culture as well as ultrasound and/or IV urography [4]. Doppler flow studies can be used as a more specific initial diagnostic tool in suspected PNCS cases. They measure the anteroposterior diameter and peak velocity at the level of the renal hilum and where the LRV crosses between the aorta and either SMA or vertebra [4]. The most common, non-invasive means of diagnosing PNCS are abdominal CT angiographic study or MRA. MRA provides a non-radiation alternative to CT. Phlebography, although more invasive, can show renocaval pressure gradients and the presence or absence of any collateral circulations [4, 11].

The management of PNCS can include surveillance, open surgery, or vascular stents placed via interventional radiology (IR). Although approximately 75% of hematuria due to NCS will resolve without intervention in the pediatric population, in patients with severe symptoms (severe pain, gross hematuria, renal insufficiency), or patients who do not improve within 24 months, stenting or surgery may be necessary. A variety of surgical approaches can be considered for patients with NCS; including nephropexy, extravascular stent implantation, transposition of the LRV (most common open technique), gonadocaval bypass, renal auto-transplantation, and nephrectomy [10]. The patient presented in this case underwent RLRV endovascular stent placement via IR due to recurrent severe episodes of pain. Complete resolution of symptoms was observed post-procedure. IR endovascular stenting advantages over open surgery techniques include the avoidance of long periods of renal congestion and extensive dissection and anastomosis [10-11]. Rare complications of IR techniques include stent thrombosis, migration, and restenosis [11].

## Conclusions

This case reports uniqueness lies in the atypical presentation of an already rare condition. It presents a clinical variant of an intriguing phenomenon adding to the small number of case reports previously described in the literature. As flank pain and hematuria are common presentations in both pediatrics and adolescence, RLRV and subsequently NCS can be easily missed by pediatricians who are not aware of it and if untreated can lead to devastating consequences. This case acts as a reminder for pediatricians to keep a wide scope of differentials in patients presenting with such symptoms; it delivers an opportunity for recognizing this anatomical variation and provides an outline of both diagnostic and treatment modalities available for these patients.
